# On a New Material and Process for Making Minute Anatomical Injections

**Published:** 1849-12

**Authors:** Paul B. Goddard


*On a new material and process for making* MINUTE *Anatomical Injections.* By PAUL B. GODDARD, M. D.

Having received recently from Europe some beautiful microscopic preparations, consisting of minute injections by Prof. Hyrtyl, Messrs. Hett, Dancer and Topping, I was stimulated to make an effort to obtain similar results, as they were, by far, finer than any which had been produced in this country. With the assistance of my friend Dr. Neill, demonstrator in the University of Pennsylvania, I made many experiments with variable results, but with such success as to lead to further investigation. At last I struck upon a plan which is uniformly productive of exquisitely beautiful results, and is moreover easy of application. For the purpose of you take purges and enemata to no purpose, for the constipation is unconquerable; you then send for the doctor when it is too late, and you die before you have time to call the attorney or the priest.

Now what does the necrotomist find? Not the mucous coat of the small intestines alone inflamed and mortified, but all the coats in some definite part of either large or small intestines equally melted down in one putrescent mass. This is what was formerly called enteritis, and it is called so still by us old-fashioned thinkers.* I have seen it so often, not merely from contortions of the bowels and strangulations in the mesentery or omentum, but also from cold or some unascertained causes, that I can hardly be mistaken. It is the *entérite phlegmoneuse* of the *Dict. de Médecine* where it is said "that it almost always comes from causes manifest;" and this sentiment is certainly true, but the author's "*presque toujours*" implies that it sometimes comes from causes not manifest, a fact I have certainly seen.

Late pathologists call it *peritonitis;* but this is a disease more diffused, as we may see by looking into the dissections of Broussais. Were you to open a belly with a bowel mortified by internal hernia, you would not surely call this peritonitis, when all the coats are equally affected? Peritonitis seldom ends in mortification; the viscera are glued together by inflammation and the belly is filled with water and pus.

In your enteritis, the mucous coat of the small intestines is inflamed; a state of things not easily ascertained. If the mucous coat of the colon is inflamed, you have dysentery—if the disease is fixed in that of the small intestines, you have diarrhœa; and if the whole tube of mucous membrane is inflamed, you have both diseases at once. Now Dr. R. P. Thomas opened a body for me, ten days ago, whose bowels were inflamed from the pylorus to the anus—this man in the new nomenclature might be said to have had duodeno-jejuno-ilio cæco-coli-rectitis, *alias* diarrhœa and dysentery at the same time.

* Dr. Watson has the same view of this subject.

making such an injection, the anatomist must provide himself with a small and good syringe; some vermillion *very finely* ground in oil;* a glass stoppered bottle, and some sulphuric ether. The prepared vermillion paint must be put into the ground stoppered bottle, and about twenty or thirty times its bulk of sulphuric ether added; the stopper must then be put in its place and the whole well shaken. This forms the material of the injection. Let the anatomist now procure the organ to be injected, (say a sheep's kidney, which is very difficult to inject in any other way, and forms an excellent criterion of success), and fix his pipe in the artery, leaving the *vein open.* Having given his material a good shake, let him pour it into a cup and fill the syringe. Now, inject with a *slow*, *gradual* and *moderate* pressure. At first, the matter will return by the vein colored, but in a few moments this will cease, and nothing will appear except the clear ether which will distil freely from the patulous vein. This must be watched, and when it ceases the injection is complete. The kidney is now to be placed in warm water of 120° Fahrenheit, for a quarter of an hour, to drive off the ether, when it may be sliced and dried, or preserved in alcohol, Goadby's solution, or any other anti-septic fluid. For glands, as the kidney, liver, &c., it is better to dry and mount the sections in Canada balsam; but for membranous preparations, stomach, intestine, &c., the plan of mounting in a cell, filled with an anti septic solution is preferable.

* That which I have used was obtained already prepared in tin tubes, at J. W. Williams', No. 37 North Sixth Street, who has obligingly assisted me to obtain the finest colors.

